# Supplementary material for: Fluid ejections in nature
Source: ArXiv. 2024 Mar 4:arXiv:2403.02359v1. Preprint. [Version 1] (PMC10942486)
Supplement: 1 [file NIHPP2403.02359v1-supplement-1.pdf]

arXiv:2403.02359v1 [q-bio.QM] 4 Mar 2024

Supplementary Information: Fluid ejections in nature

Elio J. Challita,[1,2] Pankaj Rohilla,[1] and M. Saad Bhamla[3]

[1]School of Chemical & Biomolecular Engineering, Georgia Institute of Technology, 311 Ferst Drive NW, Atlanta, GA, 30332, USA; email: saadb@chbe.gatech.edu
[2]George W. Woodruff School of Mechanical Engineering, Georgia Institute of Technology, 801 Ferst Drive NW, Atlanta, GA, 30318, USA

Annual Review of Chemical and Biomolecular Engineering 2023. AA:1–7

This article's doi:
10.1146/((please add article doi))

Copyright © 2023 by Annual Reviews.
All rights reserved

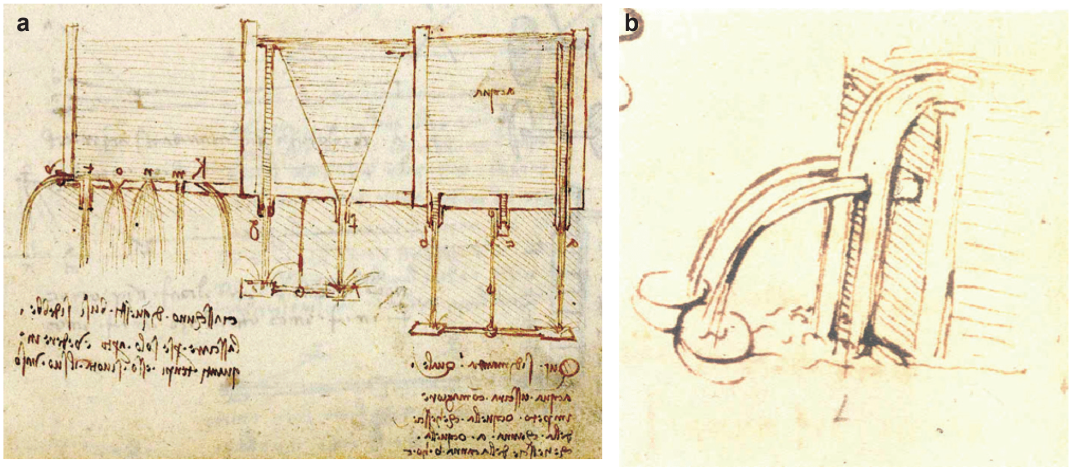

Figure S1

Leonardo Da Vinci's drawings. (*a*) "What water will pour with the greatest violence, that from the nozzle a, b or c?" - Leonardo Da Vinci (Reproduced from the Codex - National Library of Spain) (*b*) Sketch from Leonardo's Codex on the impact of jets (1, 2, 3)

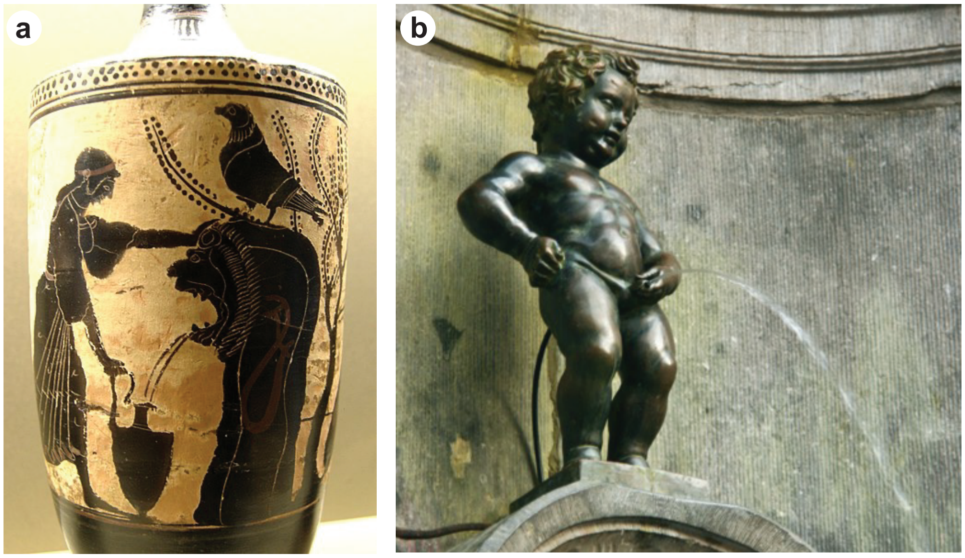

Figure S2

Historical Fountains. (*a*) Attic Greek vases from South Italy: Polyxena filling a vessel from a fountain, dated around 480 BC (Picture Source: Wikimedia, Location: The Louvre Museum, Room 652), (*b*) Mannekin Pis, a bronze statue of a urinating naked young boy (Location: Brussels, Belgium).

S1. Historical use of fluid ejections: Water Fountains

Throughout history, water ejection systems, such as fountains, have been used as water source for drinking, washing, and bathing in cities (4, 5, 6, 7, 8). Initially, these fountains relied on gravity to create liquid jets through a narrow channel. As time passed, mechanical pumps replaced gravity as

the driving force of fountains to generate powerful jets. Over time, fountains have become tourist attractions; worldwide famous example of such fountains is Manneken Pis (Brussels, Belgium) (9). Fountains have been a constant witness to the advancement of science, which has enabled us to understand how to leverage the design and power to control the fluid dynamics for required applications.

S2. Active and Passive Mechanisms in Fluidic Ejection: Dynamics, Control, and Functionality

Fluid ejection systems in nature, can be broadly categorized into active and passive mechanisms, each influencing the transient and steady-state dynamics of the fluids and setting the limits for the exerted pressure forces.

Active systems depend on muscle-powered actions, often linked to factors such as skeletal muscle force-distance relationships or the elasticity of the nozzle or pipe. Some active systems might even utilize external forces to induce a pressure differential, as seen in the milking of a cow.

In contrast, passive systems leverage external or surface forces, gravity, or osmosis to drive fluid flow, without the need for muscular action.

Control over the ejected fluid is another vital consideration. Organisms may need to manipulate the shape, speed, and breakup of the ejected fluid to meet specific functional needs such as hunting, defense, or predator avoidance. Accurate prey targeting demands high-speed fluid movement, while rapid discharge of large volumes requires maximizing the flow rate. Conversely, slow and controlled fluid release may facilitate thermoregulation through evaporative cooling. Additional complexities, such as fouling or pheromone accumulation from localized fluid concentrations, further underscore the intricate control required in fluid ejection.

S3. Jet stability

The stability of a liquid jet can be determined by different regimes within the plot of Ohnesorge number and Reynolds number. As the surrounding gas' inertial force reaches 10% of the surface tension force, the jet becomes unstable. This is marked by the onset of the "*Rayleigh break-up regime*" ($We_L > 8$ or $We_g < 1.2 + 3.41Oh^{0.9}$) beyond which the "first wind-induced" regime exists ($We_L > 8$ or $We_g < 1.2{+}3.41Oh^{0.9}$) (*Figure S3*) (10). The other two regimes of jet breakup include the second wind-induced regime ($13 < We_g < 40.3$), and the atomization regime ($We_g < 40.3$). For simplicity, the first wind-induced regime, the second wind-induced regime, and the atomized regime are merged into a single "*Other regimes of jetting*" to show the existence of continuous jetting with instabilities. The review pertains to demarcating the dripping vs. jetting in nature, and the figure presented here (*Figure S3*) captures the essence of it.

As evident in this Figure S3 and Figure 3, ballistospores, sharpshooters, aphids, mosquitoes, flies and rats eject fluid in the form of droplets. The plot also suggests that the jets of some mammals (cats, humans, horses, cats and dogs), bees, butterfly, and cicada are unstable and break up. However, fungal pores, elephants, cobras, archerfish, and cows exhibit continuous jetting. It should be noted that the cobras and archerfish jets also break up, the deviation from the Rayleigh breakup regime could be due to variations in velocity of the jet.

S4. Other fascinating examples of Fluid ejections

Some fascinating examples of fluid ejections that are not discussed in the review are presented in Figure S4. Due to limitations in data collection, some of these systems have not been studied.

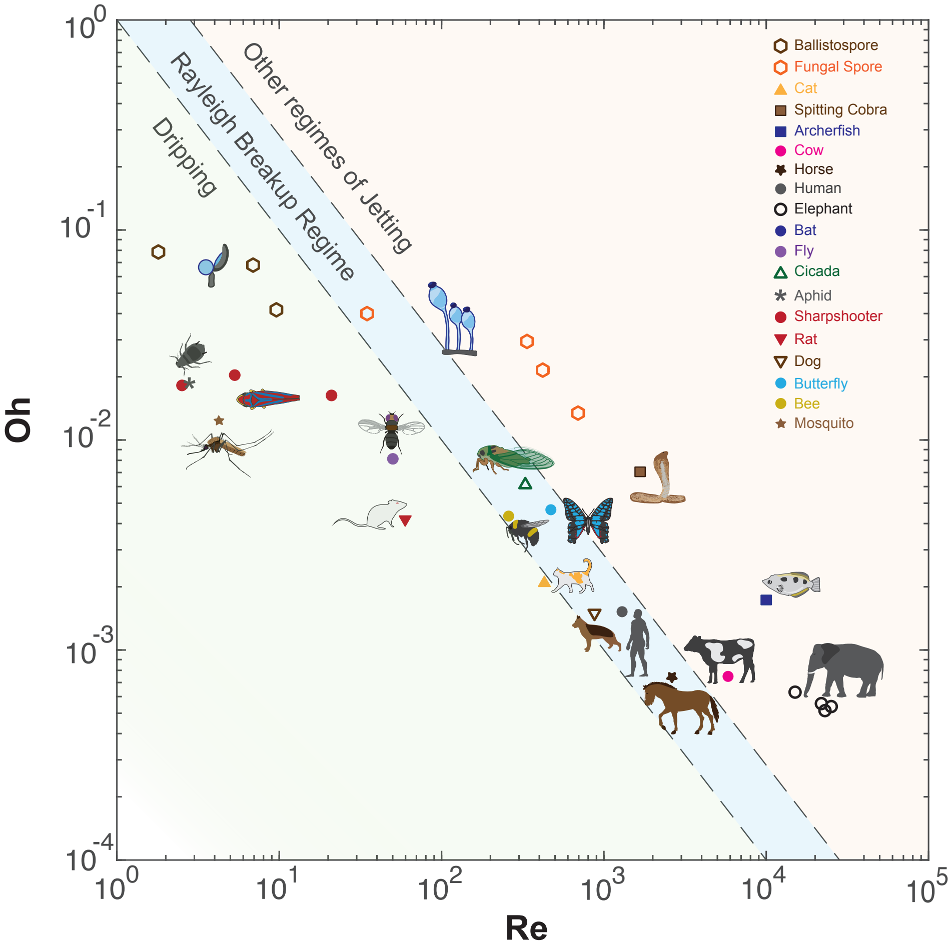

Figure S3

Ohnesorge number (Oh) vs. Reynolds number (Re) for the fluid ejections in the organisms considered in the Newtonian framework. The dripping regime is limited to $We < 1$ beyond which jetting occurs. The jet breaks up in droplets in Rayleigh breakup regime (see Box 2), beyond which jet is continuous, yet undergoes hydrodynamics instabilities. The last regime "Other regimes of jetting" includes other jetting regimes including first wind-induced regime, second wind-induced regime and finally atomized regime.

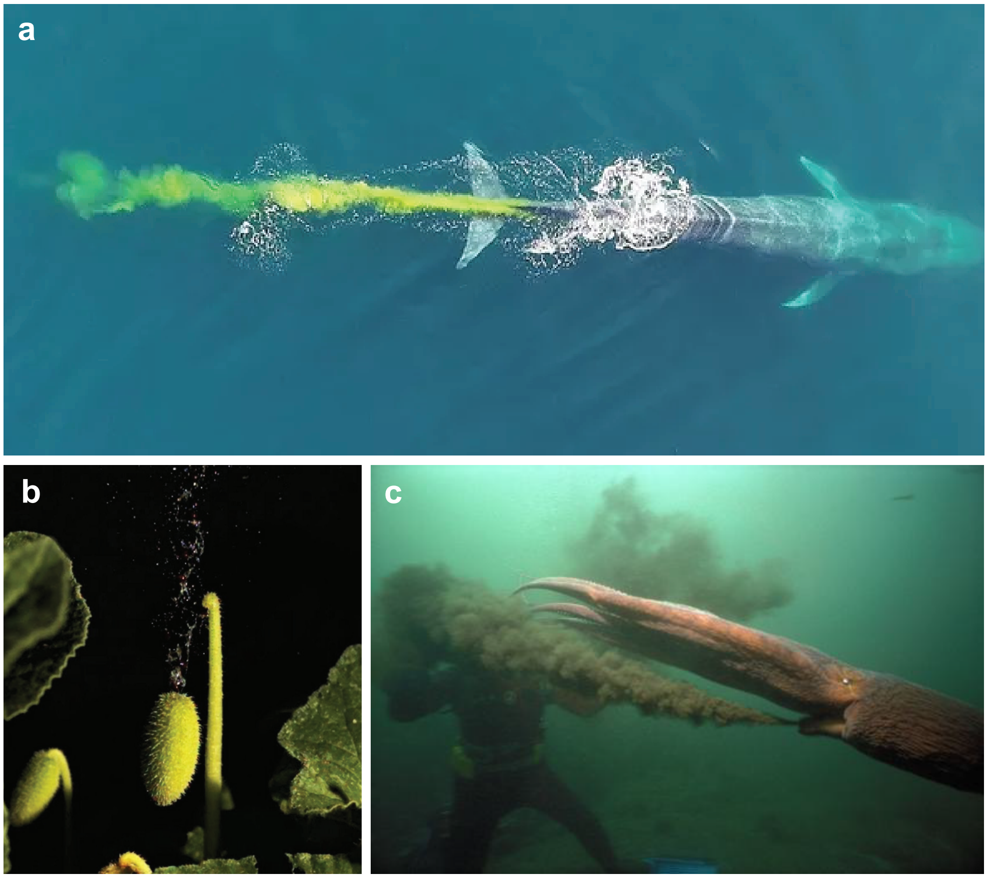

Figure S4

Some other examples of fluid ejections in organisms. (*a*) Blue whale excretion in the ocean near Perth, Australia (Credit: Ian Weise, *Permission pending*), (*b*) an octopus ejecting ink targeting a diver near British Columbia, Canada (Credit: Jeff Rotman, *Permission pending*), and (*c*) squirting cucumber (*Ecballium elaterium*) exploding cucumber seeds (Credit: Adóval Együtt)

Table 1 Table outlining the organisms, behavior, mechanism, dimensionless numbers, and sources.

Organism	Fluid	Mechanism	Behavior	Speed, u (m/s)	Orifice diameter, d (m)	Bo	We	Reference
Itersonilia perplexans	Water	Droplet, Coalescence	Spore Dispersion	1.2	8e-6	8.7e-6	0.16	(11)
Auricularia	Water	Droplet, Coalescence	Spore Dispersion	0.8	2.25e-6	6.87e-7	0.02	(11)
Sporobolomyces	Water	Droplet, Coalescence	Spore Dispersion	2.3	3e-6	1.12e-6	0.22	(11)
Ascobolus immersus	Water	Jet, Fracture-release	Spore Dispersion	14	3e-5	1.22e-4	81	(12)
Podospora anserina	Water	Jet, Fracture-release	Spore Dispersion	21	1.6e-5	3.47e-5	98	(12)
Pilobolus kleinii	Water	Jet, Fracture-release	Spore Dispersion	9	7.7e-5	8.04e-4	86.6	(12)
Basidiobolus ranarum	Water	Jet, Fracture-release	Spore Dispersion	4	8.7e-6	1.03e-5	1.93	(12)
Glassy-winged sharpshooter	Water	Droplet, Catapulting	Urination	0.4	5.2e-5	3.74e-4	0.12	(13)
Blue-green sharpshooter	Water	Droplet, Catapulting	Urination	0.16	3.3e-5	1.5e-4	0.01	(13)
Red-blue sharpshooter	Water	Droplet, Catapulting	Urination	0.06	4.2e-5	2.4e-4	0.002	(13)
Aphid	Honeydew	Droplet, Hydrophobic Waxing	Urination	0.07	4e-5	3.12e-4	0.003	https://tinyurl.com/279n6bb6
Mosquito	Blood	Droplet, Evaportation/Squeeze	Thermoregulation	0.04	1e-4	0.0014	0.0022	https://tinyurl.com/37a6b88j
Fly1	Assumed water	Droplet, Squeezing	-	0.24	1e-4	0.0014	0.079	https://tinyurl.com/mvjdxpuu
Fly2	Asummed water	Droplet, Squeezing	-	0.12	1.2e-4	0.002	0.0239	https://tinyurl.com/54a697z2
Cicada	Water	Jet	Urination	0.9	3.64e-4	0.02	4.1	This work
Butterfly	Assumed water	Jet	-	0.72	6.47e-4	0.06	4.67	https://tinyurl.com/4dw4fb8b
Bumblebee	Assumed water	Jet	-	0.37	7.4e-4	0.07	1.45	https://tinyurl.com/y5hdmfb4
Bat	Urine	Droplet	Urination	0.1	1e-3	0.13	0.14	https://tinyurl.com/4re6vk99
Wister Rat	Urine	Droplet	Urination	0.34	8e-4	0.09	1.28	(14)
Cat Female 1	Urine	Jet	Urination	0.5	0.0032	1.39	11	(14)
Cat Female 2	Urine	Jet	Urination	0.36	0.033	1.48	6	(14)
Dog	Urine	Jet	Urination	0.7	0.006	4.88	40	(14)
Human (Man)	Urine	Jet	Urination	0.7	0.007	7.23	50	(14)
Human (Woman)	Urine	Jet	Urination	0.87	0.006	4.88	63	(14)
Human (Old man)	Urine	Jet	Urination	0.6	0.006	4.88	30	(14)
Horse	Urine	Jet	Urination	0.21	0.03	122	19	(14)
Mini Horse	Urine	Jet	Urination	0.15	0.015	30	4.6	(14)
Cow 1	Urine	Jet	Urination	1.16	0.025	85	465	(14)
Cow 2	Urine	Jet	Urination	1.17	0.025	84	473	(14)
Elephant 1	Urine	Jet, fluid sheet	Urination	0.43	0.045	274	115	(14)
Elephant 2	Urine	Jet, fluid sheet	Urination	0.48	0.05	374	167	(14)
Elephant 3	Urine	Jet, fluid sheet	Urination	0.52	0.035	166	130	(14)
Asian Elephant	Urine	Jet, fluid sheet	Urination	0.44	0.048	312	128	(14)
Spitting Cobra	Venom	Jet	Spitting. defense	5	52.84e-4	0.01	97	(15)
Archerfish	Water	Jet	Spitting, predation	2	0.005	3.4	280	(16)

LITERATURE CITED

1. Leonardo Da Vinci and Owen Gingerich. *Codex leicester*. Schirmer/Mosel, 1999.
2. Leonardo Da Vinci. *The notebooks of Leonardo da Vinci*, volume 1. Courier Corporation, 2012.
3. Jens Eggers and Emmanuel Villermaux. Physics of liquid jets. *Reports on progress in physics*, 71(3):036601, 2008.
4. Said Shakerin. Art and science of water fountains. 2000.
5. Jacco H Snoeijer and Ko van der Weele. Physics of the granite sphere fountain. *American journal of physics*, 82(11):1029–1039, 2014.
6. Petri S Juuti, Georgios P Antoniou, Walter Dragoni, Fatma El-Gohary, Giovanni De Feo, Tapio S Katko, Riikka P Rajala, Xiao Yun Zheng, Renato Drusiani, and Andreas N Angelakis. Short global history of fountains. *Water*, 7(5):2314–2348, 2015.
7. Said Shakerin. Water fountains with special effects: although they were likely invented just to deliver water, fountains became much more than reservoirs early in human history. *American Scientist*, 93(5):444–452, 2005.
8. Ari Hynynen, Petri Juuti, Tapio Katko, et al. *Water fountains in the worldscape*. International Water History Association and KehräMedia Inc., 2012.
9. Catherine Emerson. *Regarding Manneken Pis: Culture, Celebration and Conflict in Brussels*. Routledge, 2017.
10. S. P. Lin and R. D. Reitz. Drop and spray formation from a liquid jet. *Annual Review of Fluid Mechanics*, 30(1):85–105, 1998.
11. Anne Pringle, Sheila N Patek, Mark Fischer, Jessica Stolze, and Nicholas P Money. The captured launch of a ballistospore. *Mycologia*, 97(4):866–871, July 2005.
12. Levi Yafetto, Loran Carroll, Yunluan Cui, Diana J Davis, Mark WF Fischer, Andrew C Henterly, Jordan D Kessler, Hayley A Kilroy, Jacob B Shidler, Jessica L Stolze-Rybczynski, et al. The fastest flights in nature: high-speed spore discharge mechanisms among fungi. *PLoS One*, 3(9):e3237, 2008.
13. Elio J Challita, Prateek Sehgal, Rodrigo Krugner, and M Saad Bhamla. Droplet superpropulsion in an energetically constrained insect. *Nature Communications*, 14(1):860, 2023.
14. P. J. Yang, J. Pham, J. Choo, and D. L. Hu. Duration of urination does not change with body size. *Proceedings of the National Academy of Sciences*, 111(33):11932–11937, 2014.
15. Bruce A Young, Karen Dunlap, Kristen Koenig, and Meredith Singer. The buccal buckle: the functional morphology of venom spitting in cobras. *Journal of Experimental Biology*, 207(20):3483–3494, 2004.
16. Alberto Vailati, Luca Zinnato, and Roberto Cerbino. How archer fish achieve a powerful impact: Hydrodynamic instability of a pulsed jet in toxotes jaculatrix. *PLOS ONE*, 7(10):1–8, 10 2012.
